# Exploring Modeling Techniques for Soft Arms: A Survey on Numerical, Analytical, and Data-Driven Approaches

**DOI:** 10.3390/biomimetics10020071

**Published:** 2025-01-24

**Authors:** Shengkai Liu, Hongfei Yu, Ning Ding, Xuchun He, Hengli Liu, Jun Zhang

**Affiliations:** 1 Shenzhen Institute of Artificial Intelligence and Robotics for Society, Shenzhen 518000, China; dingning@cuhk.edu.cn (N.D.); hexuchun@cuhk.edu.cn (X.H.); liuhengli@cuhk.edu.cn (H.L.); zhangjun1@cuhk.edu.cn (J.Z.); 2 School of Data Science, The Chinese University of Hong Kong, Shenzhen 518172, China; hongfeiyu@link.cuhk.edu.cn

**Keywords:** soft arms, modeling approaches, numerical methods, analytical techniques, data-driven models

## Abstract

Soft arms, characterized by their compliance and adaptability, have gained significant attention in applications ranging from industrial automation to biomedical fields. Modeling these systems presents unique challenges due to their high degrees of freedom, nonlinear behavior, and complex material properties. This review provides a comprehensive overview of three primary modeling approaches: numerical methods, analytical techniques, and data-driven models. Numerical methods, including finite element analysis and multi-body dynamics, offer precise but computationally expensive solutions for simulating soft arm behaviors. Analytical models, rooted in continuum mechanics and simplified assumptions, provide insights into the fundamental principles while balancing computational efficiency. Data-driven approaches, leveraging machine learning and artificial intelligence, open new avenues for adaptive and real-time modeling by bypassing explicit physical formulations. The strengths, limitations, and application scenarios of each approach are systematically analyzed, and future directions for integrating these methodologies are discussed. This review aims to guide researchers in selecting and developing effective modeling strategies for advancing the field of soft robotic arm design and control.

## 1. Introduction

Soft arms have emerged as a transformative technology, drawing inspiration from the versatility and adaptability of biological organisms. These bionic systems replicate the natural flexibility, dexterity, and safety found in animals like octopuses, enabling a wide range of applications in fields such as industrial automation, medical assistance, and exploration in unstructured environments. Unlike traditional rigid robots, soft robotic arms leverage deformable materials and compliant structures to achieve complex movements and interactions. However, the unique characteristics of these systems—such as nonlinear dynamics, high degrees of freedom, and intricate material behaviors—pose significant challenges for accurate modeling and control. To address these challenges, the development of robust and efficient modeling methods is crucial for advancing the design, optimization, and practical deployment of soft robotic systems.

Building upon these challenges, various modeling approaches have been proposed to describe and predict the behavior of soft arms. Broadly, these approaches can be categorized into numerical [1], analytical [2], and data-driven methods [3], each offering unique strengths and limitations. Numerical methods, such as finite element analysis (FEA) [4,5], provide highly accurate simulations by discretizing the soft arm’s structure and solving its governing equations. However, these methods are computationally expensive, limiting their application in real-time control scenarios.

Analytical models [6,7], on the other hand, aim to derive simplified equations based on physical principles, such as continuum mechanics or beam theory. While these models are computationally efficient, they often require assumptions that may oversimplify the system’s dynamics, particularly in complex configurations.

Recent advancements in artificial intelligence have introduced data-driven approaches, leveraging machine learning algorithms to model soft robotic arms directly from experimental data [8,9,10]. These methods bypass the need for explicit physical modeling, enabling adaptive and flexible solutions. However, their reliance on large datasets and generalization capabilities remains a topic of ongoing research.

This review aims to provide a comprehensive overview of three modeling approaches for soft robotic arms, focusing on their principles, applications, and integration prospects. These modeling methods not only draw inspiration from the movement patterns and control mechanisms of biological organisms but also integrate bionic principles into the design and control of soft robots. By critically analyzing the strengths and limitations of each approach, we will explore their potential and constraints under bionic inspiration. The goal of this review is to offer valuable guidance for future research, advancing more effective and innovative modeling strategies for soft robotic arms, particularly in terms of bionic design and control optimization, to meet the increasingly complex demands of real-world applications.

## 2. Analytical Solution Model

The analytical solution model represents a powerful approach in the modeling of soft robotic systems, offering a balance between computational efficiency and physical interpretability. Unlike numerical methods, which rely on discretization and complex computations, analytical models attempt to derive closed-form solutions that describe the behavior of the system using simplified physical principles and assumptions. These models are particularly useful when dealing with relatively simple geometries or when an understanding of the underlying mechanics is crucial. For soft robotic arms, analytical models often draw from concepts in PCC (piecewise constant curvature) model, beam theory, and elastodynamics, providing insights into key performance metrics such as displacement, strain, and stress. Although they may not capture the full complexity of real-world soft robotics, analytical solutions are valuable for providing quick, efficient predictions and for gaining a deeper understanding of the fundamental dynamics at play. This makes them an essential tool in the early stages of design and optimization, where rapid prototyping and system analysis are required.

### 2.1. Euler–Bernoulli Beam Theory

**Figure 1:**
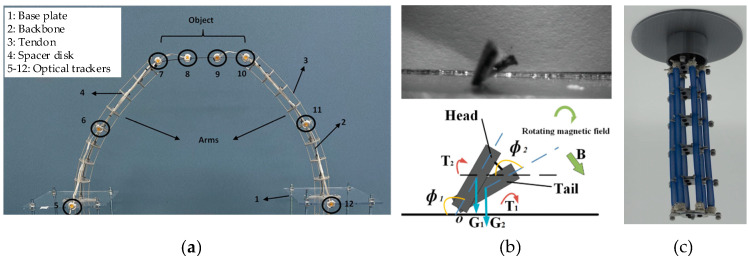
Part of a soft robot modeled using Euler–Bernoulli beam theory: (**a**) tendon-driven soft arm [17]; (**b**) micro soft robots based on magnetoelastic composites [18]; (**c**) pneumatic soft arm [19].

The Euler–Bernoulli beam theory assumes that the cross-section of the beam has infinite stiffness in its own plane and remains plane and perpendicular to the axis of the beam after deformation during deformation [11]. As shown in Figure 1, this theory is widely used in studying the bending behavior of beams. For a beam with elastic modulus E and section moment of inertia I when a distributed load q is applied to it, the relationship between the load and the induced deflection ω(x) can be described by the following equation:

(1) $$\frac{d^{2}}{dx^{2}}\left(EI\frac{d^{2}\omega }{dx^{2}}\right)-q\left(x\right)=0$$

and supplemented by specific boundary conditions [11]. This theory is widely used to analyze the bending behavior of beams under various loading conditions.

Euler–Bernoulli beam theory is often used to describe continuous bending deformation. For example, in studying the dynamics of fluid elastic actuator fingers, Mbakop [12] used this theory to develop an inverse dynamics model based on a three-dimensional Euler–Bernoulli beam, and on this basis derived a modular dynamics model for a cable-driven soft robot [13]. In addition, the Euler–Bernoulli beam method has also been used in the analysis of classical soft robots. Through linear beam theory, Coulomb friction law and simplified energy analysis, the relationship between bending curvature, bending stiffness and air pressure was established [14]. In control and interaction modeling, the effect of external forces is a factor that needs special consideration. For example, a simplified solid mechanics model can capture soft body deformation, and the peeling and loading mechanism of unconstrained soft robots on three-dimensional surfaces can be studied by controlling the external magnetic field [15]. Although the simulation accuracy of such discrete models may not be as high as other general simplified models, their computational efficiency is significantly improved and computational time is saved [16].

When the application scenario requires higher accuracy, the Euler–Bernoulli beam model can also be further extended. For example, the piecewise constant curvature model was successfully applied to the dynamic simulation of flexible links by combining this theory, thereby effectively improving the accuracy of modeling [20].

### 2.2. PCC Model

**Figure 2:**
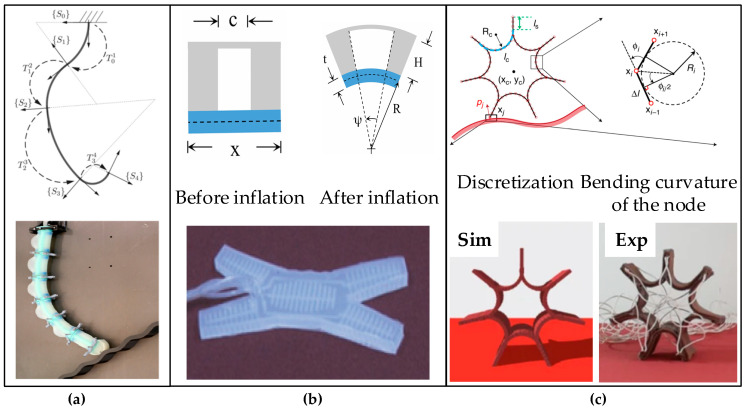
Three soft robots modeled using the PCC model: (**a**) Kinematic model of a four-segment soft robot based on constant curvature [38]; (**b**) Soft quadruped robot [39]; (**c**) Discretize the flexible rolling robot using a discrete model and calculate the bending curvature [40].

The PCC model has been widely used in modeling continuum robots (as shown in Figure 2). The model assumes that the soft body is represented by a finite number of arcs, each with a constant curvature in space. This simplification greatly reduces the complexity of calculating the bending angle of the soft arm and makes kinematic modeling easier to implement. PCC models can be divided into kinematics-based models and mechanics-based models [21].

#### 2.2.1. Kinematics-Based PCC Model

If the continuum is represented as a finite CC (constant curvature) segment, each CC segment can be represented by a finite arc parameter. In PCC, the curvature κ, the angle of the plane containing the arc φ, and the arc length s are three key parameters used to characterize the robot.

In the context of infinite-dimensional soft robots, the homogeneous transformation matrix of any point along the arc can be determined starting from the base point of the arc. This parameterization, combined with the PCC assumption, enables a more efficient kinematic model. In addition, many modeling methods and control strategies originally developed for rigid robots can also be directly applied to soft robots. For example, Equation (2) shows a homogeneous transformation matrix derived based on the Denavit–Hartenberg parameterization method [22].

(2) $$T\left(\kappa ,\phi ,s\right)=\left[\begin{array}{cccc} c\phi c\kappa s & -s\phi & c\phi s\kappa s & \frac{1}{\kappa }c\phi \left(1-c\kappa s\right)\\ s\phi c\kappa s & c\phi & s\phi s\kappa s & \frac{1}{\kappa }\left(1-c\kappa s\right)\\ -s\kappa s & 0 & c\kappa s & \frac{1}{\kappa }s\kappa s\\ 0 & 0 & 0 & 1 \end{array}\right]$$

One of the main limitations of the PCC model is that when the curvature approaches zero, its parameterization and kinematic diagram may implicitly induce numerical singularities, causing the radius of curvature to approach infinity or be undefined. To solve this problem, researchers have proposed a variety of solutions.

References [23,24] use modal form to represent the rotation and position components of the homogeneous transformation matrix, and numerically approximate the actuator length variable near zero through Taylor series expansion. Based on this method, references [25,26] proposed a Lagrangian method for the spatial dynamics of a single-segment continuous arm for underwater operations. This method takes into account a variety of physical factors and mechanical constraints and has high accuracy. At the same time, the author further extended this modeling method to multi-segment arms in reference [27]. References [28,29] proposed an alternative method based on exponential mapping, and the secondary transformation matrix formula can be derived through exponential coordinates. Reference [30] assumes that the two concentric tubes have the same stiffness and are torsionally rigid, and apply uniform torque to each other. Based on the arc assumption, the Euler–Bernoulli beam linear equation is used to establish the bending model in the plane, and the final curvature of the two overlapping tubes is calculated by a force balance formula similar to that of parallel linear springs.

Similarly, in reference [31], the parameterization of the arc is represented by axis rotation, thus avoiding the singularity problem common in previous methods. This method is computationally efficient and does not depend on the number and position of actuators (such as tendons or artificial muscles), making real-time dynamic modeling of manipulators composed of continuous joints possible. Reference [32] proposed a method based on screw theory, which further provided a mapping between configuration parameters and the length of the driving tendon.

Reference [33] proposed another parameterization method that avoids singularities near the zero curvature configuration. For each part of the robot, the configuration of the segment is described by a linear combination as the superposition of four arcs contained in the volume of the part. In particular, considering the length difference between adjacent arcs and combining the geometric relationship, the explicit expression of the homogeneous transformation matrix under the new parameters can be obtained by algebraic derivation.

#### 2.2.2. PCC Model Based on Mechanics

Some researchers have tried to derive the mechanical response of a soft arm by applying static laws to its shape described by PCC. Reference [34] derived the forward and inverse kinematics of a tendon-driven manipulator by applying static laws to the morphological description of the soft arm. Subsequently, the model was extended to the case of a finite number of tendons and verified in a set of parallel linear spring systems. In reference [35], the authors further developed a three-dimensional static model. Reference [36] is one of the earliest papers to combine the PCC model with the Euler–Lagrange equations. In this paper, the authors combined the mechanical interconnection of the parallel and the transmitted bellows and solved the dynamic model of the space continuous manipulator using the analytical derivatives of constant curvature kinematics. The dynamic model obtained using this method can be used to simulate the manipulator dynamics and also to calculate the inverse dynamics required for model-based controller design or path planning. Furthermore, reference [37] introduces the dynamic modeling and trajectory tracking control design of a hyper-redundant continuous manipulator (HRM). The authors considered the influence of the robot’s dynamic potential energy and elastic potential energy and derived a PCC-based dynamic model using the Euler–Lagrangian method.

## 3. Numerical Solution Model

The numerical solution model has become a cornerstone in the modeling of soft robotic systems, particularly when dealing with complex geometries, nonlinear behaviors, and intricate interactions between different components. Unlike analytical models, which often rely on simplifications, numerical methods offer a more detailed and flexible approach by discretizing the system into smaller elements and solving the governing equations iteratively. Techniques such as finite element analysis (FEA), system mechanics approach, and Cosserat rod theory are widely used in soft robotics to simulate the deformation, stress distribution, and dynamic response of soft actuators and structures. These methods allow for the accurate prediction of system behavior under various loading conditions, making them particularly valuable for design optimization, performance analysis, and control system development. While numerical models can be computationally intensive, advances in computational power and algorithmic efficiency have made them increasingly feasible for real-time applications, offering a high degree of accuracy in capturing the complexities of soft robotic behaviors.

### 3.1. FEM

Currently, three-dimensional continuous mechanical models are usually established using the finite element method (FEM). FEM is a widely used numerical technique for solving approximate solutions to partial differential equations (PDEs). Its core is to divide complex large systems into smaller finite element units through spatial discretization. The typical workflow of FEM includes steps such as geometry modeling, configuration and constraint definition, meshing, analysis setup, solution, and post-processing. Using FEM-based static models, the boundaries of the external workspace can be accurately visualized and estimated, thereby supporting optimization-based control [41].

The soft bellows actuator in [42] was modeled using FEM to explore the mechanical properties along different latitudes. This approach enables accurate control based on pressure and motion curves. Reference [43] shows that factors such as robot gravity, loads on actuators or motors, and contact forces with internal components or the external environment can introduce significant errors, and FEM models have been proven to be effective in dealing with these complex effects.

In addition, when accurately simulating tensegrity topologies, bending degrees of freedom and regional elongation must be considered to capture bending and contraction motion patterns. Reference [44] decomposes tensegrity structures into multiple components (such as struts, springs, and cables) and nodes. In this structure, the generalized coordinates of each element consist of the sum of two node position vectors, forming a position formula in FEM. This approach can provide higher accuracy and flexibility for modeling complex topologies.

Recently, the literature [45] proposed a method that combines the finite element method with discrete Cosserat modeling to capture the mechanics and actuation of soft robots. Instead of imposing meshing rules, the two models are linked together using kinematic constraints, and it has been shown that the forward and inverse kinematic models of the robot can be obtained through quadratic optimization.

In addition, since the standard finite element method is not differentiable at the element boundary, some researchers have proposed the moving least squares (MLS-FEM) method to make the deformation gradient have second-order differentiability [46]. This improvement aims to apply optimization techniques (such as gradient descent) to different design problems such as automatic actuation path planning of soft robots [47] and sensor design [48].

At the same time, FEM has become the core technology of many popular simulation software, such as Abaqus (version 5.4), ANSYS (version 24.2), COMSOL (version 6.3.0.290), etc. These softwares can be used as powerful general tools for a wide range of physical problems, including structural dynamics, fluid interaction, thermodynamics and other fields. At the same time, these softwares that apply FEM are now widely used in the practice of soft robot modeling, such as Abaqus in references [49,50], and ANSYS in reference [51].

In the field of soft robotics, some specially designed FEM tools and applications have also been proposed. For example, in 2007, researchers from multiple research institutions released an open source C++ library called Sofa, which is a computing environment originally developed for medical simulation [52]. Over time, Sofa has gradually developed into a comprehensive, high-performance library and has been widely used in multiple application areas. For the design, modeling and control of soft robots, Sofa has developed a dedicated plug-in SoftRobots [4], and even proposed a function to handle self-collision scenarios in the literature [53]. Open source software such as ChainQueen (version 2) [54], TMTDyn (https://github.com/smhadisadati/TMTDyn) [16], IPC (version 2) [55], AMBF (version 2) [56], and SOFiSTiK (version 2025) [57] have also enriched the community and provided researchers with high-quality FEM-based simulation platforms. Table 1 lists these FEM-based simulators and introduces their main application areas.

### 3.2. System Mechanics Approach

In the above section, the PCC model, which can directly obtain analytical solutions, is introduced. By combining the energy method with the PCC model, the mechanical equilibrium conditions of the system can be directly derived from the relationship between displacement, strain and external force, avoiding the complex differential equation solution.

In reference [58], the authors used the strain energy function based on Cauchy-Green stretching to analyze the influence of cross-sectional deformation on arm deformation for the static problem of a braided pneumatic continuum manipulator. It was found that when the PCC parameterization failed, releasing the PCC constraint could obtain a more accurate model.

However, the traditional PCC model mainly focuses on bending behavior but does not consider the torsion effect. To solve this problem, researchers have proposed several improved versions of the PCC model. In references [59,60], the dynamics of a tendon-driven continuous robot were derived using the virtual work method. This method is applicable to any type of external force or torque, including dissipative effects and external loads. The torsion model is particularly critical in the mechanical modeling of concentric tube robots. In [61], the authors first proposed the design conditions required for the effectiveness of the PCC method and established a general model of a concentric tube robot. In this model, the relative torsion angle between the two tubes is defined as a function of the arc length, which is then used to write the moment equilibrium equations and compatibility constraints to ensure the coincidence of the tube centerlines. The torsional strain equations are derived from the equilibrium equations of the Cosserat bar, and the entire set of input and output relations is explored by solving the resulting boundary value problems. Based on this model, stability analysis was further carried out in [62,63].

### 3.3. Cosserat Rod Theory

Kirchhoff rod theory is applicable to slender solids that satisfy one-dimensional geometric characteristics, that is, their length is much larger than the cross-sectional radius. This theory mainly describes the bending and torsion behavior of slender structures.

In the early 20th century, the Cosserat brothers reformulated the Kirchhoff rod theory by introducing the concept of “director” and developed the Cosserat rod theory [64]. Compared with the Kirchhoff rod theory, the Cosserat rod theory, on the basis of considering bending and torsion, further covers all possible deformation modes such as tension and shear, and is therefore regarded as an extension of the Kirchhoff rod theory.

**Figure 3:**
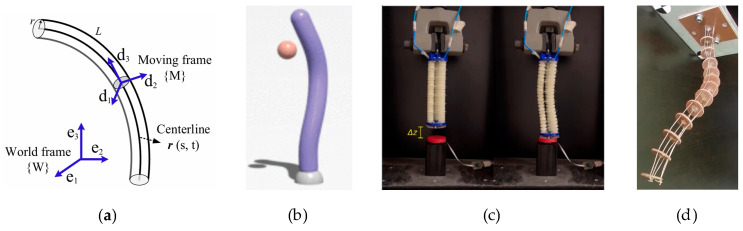
Principle, simulation, and application of Cosserat rod theory: (**a**) schematic diagram of the Cosserat rod theory; (**b**) Elastica, a soft robot simulator developed based on Cosserat rod theory; (**c**) the soft robotic arm in [70] first learns the reinforcement learning strategy in Elastica, and then applies it to the real world; (**d**) Cosserat rod theory can be used to model different types of soft robots. This is a wire-driven soft arm based on Cosserat modeling.

As shown in Figure 3a, the Cosserat bar is described by its centerline position vector r(s,t) and the local reference system $\left(d_{1},d_{2},d_{3}\right)$, where s represents the arc length of the bar, t represents time, and $d_{i}$ is the pointing vector. The strain state can be obtained by calculating the rate of change of the fixed body coordinate system relative to the arc length. The equilibrium equations of forces and moments are established by describing their changes over time. Linear constitutive relations are used to relate geometric deformations, material properties, and the corresponding internal forces and internal moments.

In order to improve the prediction accuracy, in [65], the Cosserat rod model was extended by combining it with the principle of minimum potential energy and the application of the Newton–Euler law in [66]. Based on the Cosserat rod model, [67] successfully constructed a kinematic static model of a parallel continuous robot. In [68], accurate and computationally efficient modeling of nonlinear arms was achieved by integrating screw theory, Lie groups and Lie algebras, and the finite element method. In addition, Elastica, an open source environment developed based on the Cosserat rod model, as described in [69], was used to simulate the three-dimensional dynamics of soft and slender rods, including bending, twisting, shearing, and stretching behaviors, which significantly improved the computational efficiency and was able to dynamically simulate the interaction between multiple active or passive Cosserat rods and their interaction with the environment.

Although the Cosserat rod model can improve the modeling accuracy, the large amount of computation in the solution process is a problem. To this end, Chen [71] proposed a Cosserat rod model based on shape assumptions to model multi-skeleton continuum robots, which simplifies some constraints in the modeling and greatly shortens the time for solving the inverse kinematics while ensuring accuracy. Mitros [72] developed a new numerical solver that improves the computational efficiency of the model and facilitates the deployment of the robot. Wu [73] solved the model using a variant of the Cosserat method and the Levenberg–Marquardt method, which accelerated the model solution and enabled near real-time applications of continuum robots with multiple intermediate constraints. Reference [74] proposes a Cosserat-based numerical method for real-time forward dynamics simulation. This method implicitly discretizes the inverse of the PDE and then solves the resulting arc length ODE boundary value problem at each time step. After experimental verification on robots with different drive sources (retractable rods, tendons, and fluid chambers), it is found that this method is simple to implement while having high computational efficiency and model accuracy.

### 3.4. ANCF

Shabana [75] proposed the absolute nodal coordinate method (ANCF) in 1996. This method uses position gradient coordinates to replace the angular coordinates of traditional finite elements. The system differential equation derived based on this method does not contain centrifugal force and Coriolis force, and the mass matrix is a constant matrix. Compared with other modeling methods, this method can model complex objects with a small number of units, which enables it to accurately describe flexible bodies with large rotation and large deformation. Therefore, this method is suitable for dynamic modeling of soft arms.

In recent years, modeling of soft arms based on ANCF has also become very common. In [76], the authors proposed a continuum-based method, which uses the position and position gradient of the absolute node coordinate formula to formulate rheological specified trajectory and shape constraint equations, which are used to define the driving control force, and successfully control the motion and shape of soft robots and materials. In [77], the authors proposed a variable curvature kinematic modeling method for flexible continuum robots considering external forces, which achieved precise motion control of the robot. In [78], ANCF was used to model soft parallel robots. The authors developed a continuum-based model to describe and parameterize the global configuration and large deformation of the parallel robot, and achieved good results in the tracking task of the end effector. The author further proposed a fully parameterized model in [79] to solve the modeling problem of slender pneumatic robots. This method uses Hermite interpolation to accurately describe the robot’s force components, and further derives an estimation algorithm that can infer the complete robot configuration and external force distribution from limited motion data.

### 3.5. Model Order Reduction Method

Model order reduction (MOR) is a popular technique that aims to reduce computational complexity while preserving system accuracy by constructing a model with minimal degrees of freedom. Most MOR methods obtain data by calculating full-order model snapshots under different parameters.

Among these MOR methods, orthogonal decomposition is widely used in. In addition, proper orthogonal decomposition (POD) reduces the amount of computation and retains the main dynamic characteristics by using two orthogonal projection operators to decompose high-dimensional vectors into reduced-order states and neglected states [80]. Reference [81] also achieved good results by appropriately modifying the projection operator of proper orthogonal decomposition to maintain the stability and mechanical structure characteristics of the system. However, when there is intermittent contact in the system, the contact area or contact position may change. In this case, due to the lack of intermediate mechanical variables, the previous MOR method may no longer be applicable. To address this problem, the balanced model order reduction method becomes particularly effective, which allows switching between multiple reduced-order models to adapt to contact and changing conditions [82]. In the self-collision problem, the MOR method effectively reduces the dimension of the contact force space by projecting the contact force onto the reduced positive space. In addition, a data-driven hybrid MOR strategy was proposed in [83], which combines symplectic decomposition and orthogonal decomposition techniques to derive smooth forces and non-smooth forces, respectively. Experimental results show that the efficiency of the hybrid MOR strategy is improved by 65.89%. In [84], the authors further considered the cable constraints and friction effects based on the virtual power principle and established a real-time dynamic model for a cable-driven continuous robot.

## 4. Data Driven Modeling

Data-driven modeling has emerged as a significant advancement in the field of soft robotics, leveraging experimental data and machine learning techniques to directly learn and predict system behaviors without relying on traditional physical modeling. Unlike analytical and numerical models, which depend on explicit physical assumptions, data-driven models uncover hidden patterns and relationships in system behavior by analyzing large amounts of real-world data. This approach is particularly advantageous for dealing with complex, nonlinear soft robotic systems, especially in dynamic environments or unknown conditions where traditional models may struggle. Common data-driven modeling techniques include deep learning, regression analysis, and support vector machines, which can automatically extract features from data and make predictions. While data-driven methods typically require large, high-quality datasets, their flexibility and adaptability make them highly promising for applications in real-time control, adaptive modeling, and optimization.

In the previous chapters, we discussed the modeling methods based on analytical solutions and numerical solutions, respectively, which laid a solid theoretical foundation for the modeling of complex systems. However, with the improvement of data acquisition and processing capabilities, data-driven modeling methods have gradually shown great potential. This type of method uses data obtained from external sources to model soft robots, which can avoid solving complex model solutions.

### 4.1. Statistic Method

**Figure 4:**
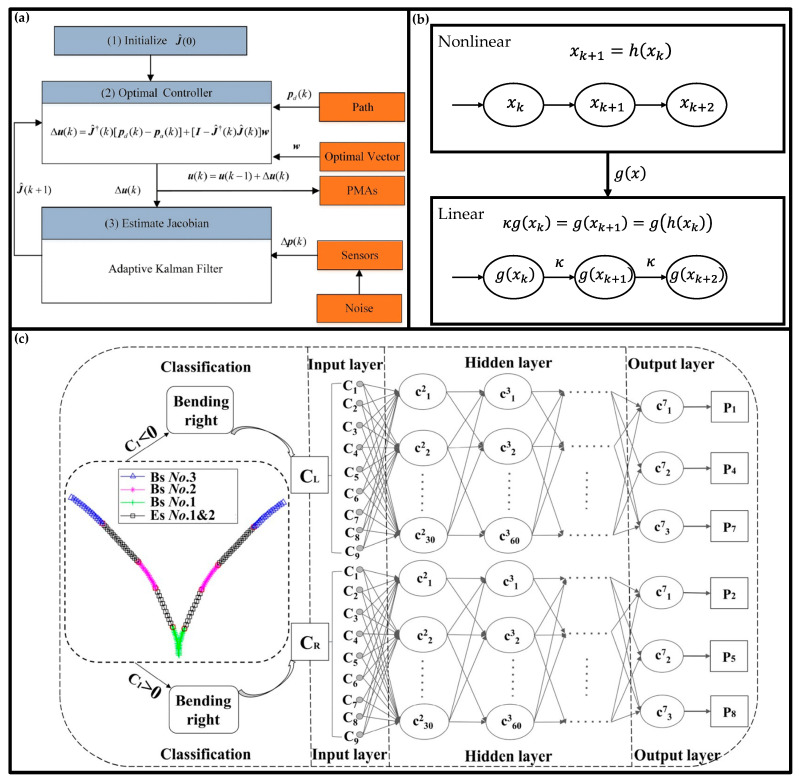
Schematic diagram of some typical statistical methods: (**a**) modeling and controlling a soft arm using a Kalman filter; (**b**) Koopman operator theory can establish a mapping from nonlinear systems in low-dimensional space to linear systems in high-dimensional space; (**c**) modeling a soft arm using a combination of multilayer perceptron and regression methods [90].

Statistical methods do not rely on the physical relationship between variables, but use statistical methods to directly establish mapping relationships between variables. In soft robot modeling, traditional machine learning methods have been used for a long time, many statistical regression methods have been widely used, such as linear regression [85], support vector regression [86], Gaussian process regression [87], etc. In addition to regression methods, Gaussian mixture models [88] are also often used to summarize the collected data, and extended Kalman filters [89] (as shown in Figure 4a) are often used as observers to estimate the robot state.

Although these traditional machine learning methods can effectively capture the relationship between soft robot variables, they usually rely on large-scale training data and are difficult to directly reflect the physical properties of the system. In order to make up for this deficiency, modeling methods based on Koopman operator theory have gradually attracted attention in recent years. Koopman operator theory maps nonlinear dynamic systems to high-dimensional linear spaces, providing an analytical linear representation while retaining the dynamic characteristics of the system [91]. This method can combine the advantages of data-driven technology and physical modeling, thus showing great potential in the complex dynamics modeling of soft robots.

Koopman operator theory was applied to the model-based control of the Sphero SPRK robot in 2017. Later, Daniel Bruder et al. discovered the advantages of Koopman operator theory and became one of the first researchers to use this theory to model soft robots. In [92], the author introduced a method based on Koopman for system identification and construction of a pneumatic flexible robot arm model, and used a model predictive controller (MPC) for trajectory tracking control, achieving good results. In [93], the author introduced the concept of static operator as the key pre-increment of optimal control based on Koopman operator theory, which can accurately model soft robotic arms and take inertia into account, and promote the rapid modeling and control of soft robots from quasi-static to inertial fields. In [94], researchers established a model of bronchoscopic robot for lung intervention surgery based on Koopman operator, and proposed a new MPC-based flexible pipe bending control method, which significantly improved the reliability and accuracy of tracking tasks. In [95], the author proposed a more data-efficient modeling method. This method first identifies the physical Koopman model and then supplements the model with a data-driven residual model, thereby obtaining a model that is more accurate and requires less data than a pure physical model. In [96], the external load is incorporated into the Koopman operator model as a state. Using this method, the size of the external load can be estimated online by the observer, and trajectory tracking can be accurately achieved.

### 4.2. Neural Network Method

**Figure 5:**
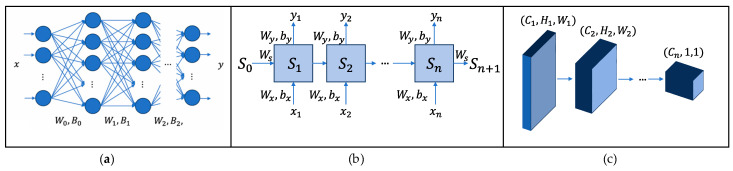
Schematic diagram of partial neural network method: (**a**) MLP, which consists of multiple layers, and the parameters $W_{i},\;W_{i}$ in a layer are parallel and can be trained simultaneously; (**b**) RNN, which takes input data x sequentially, and each bar shares the same weights $W_{S},\;W_{x},\;W_{y},\;b_{x},\;b_{y}$; (**c**) CNN, which takes a matrix with channel $C_{1}$, height $H_{1}$ and width $W_{1}$ as input, and finally CNN outputs a matrix with dimension $\left(C_{n},\;1,\;1\right)$.

Neural network (NN) is a powerful tool that has been widely used to solve nonlinear problems in many fields, including robotics. Its design is inspired by biological neural networks, which are the nervous systems that control the functions of animal brains. Artificial neural networks simulate the behavior of biological neurons and transmit signals between neurons to form complex information processing capabilities. In artificial neural networks, neurons are the basic building blocks of the network. Each neuron receives input signals (usually real numbers) from other neurons and combines these signals through nonlinear functions to generate outputs. Neurons in the network interact with each other through connected edges, and the weight of each edge is constantly adjusted during training to optimize network performance. These neurons are usually organized in a hierarchical manner: the first layer is called the input layer, which is responsible for receiving external data; the last layer is the output layer, which generates the prediction results of the model; and the hidden layer in between is responsible for extracting the deep features of the data. Through the interaction of multiple layers of neurons, neural networks construct a set of highly complex and nested nonlinear functions that can efficiently capture complex patterns and relationships in data, thus becoming a core tool for solving diversity problems. Figure 5 shows the principles of some commonly used neural networks.

The method of using a neural network algorithm to control continuum robots was first proposed in the literature [97]. Specifically, the controller compensates for the dynamic uncertainty of the system through a fuzzy neural network, thereby effectively reducing the range of uncertainty.

In [98], the researchers used multilayer perceptron (MLP) and radial basis function (RBF) neural network in a fuzzy neural network to model the forward kinematics of the bionic power-assisted trunk. The input layer of the neural network is responsible for receiving the input variables and propagating them to the hidden layer, and each neuron in the hidden layer is associated with an RBF kernel (usually a Gaussian kernel). The experimental data were collected by trilateration algorithm, recording the position of the arm tip under different driving pressures.

In another study [99], the author combined data-driven methods and pseudo-primitive modeling to model the bionic power-assisted trunk. Pseudo-primitive modeling regards the trunk as a sequence of rigid vertebrae connected by four prismatic joints, while data-driven modeling uses a modified Elman neural network. Cecilia et al. [100] proposed a supervised learning method using a Jacobian matrix and feedforward neural network-based approach to solve the inverse statics problem of non-constant curvature soft manipulators.

These studies use kinematic models to describe the configuration of the robot, while the dynamic model establishes the connection between the robot motion and the driving force. The direct dynamic model is constructed based on the geometrically accurate model [101], and the input of the fuzzy neural network is the position of the rope tip and the output is the rope tension. In the experiment, the position of the rope tip was obtained through an infrared vision system, and the fuzzy neural network was optimized and trained with a set of tension data. After the training was completed, the output of the fuzzy neural network was used as the input of the direct dynamic model, and its performance was verified on the test set. The authors further proposed a machine learning approach in reference [102], applying a unique formulation to introduce fuzzy neural networks into the modeling and closed-loop kinematic control of continuum robots.

In [103], the authors proposed a dynamic model of a soft robotic arm for open-loop control. Under the assumption that the robot and the task space have the same degrees of freedom, the forward dynamics equations are expressed solely in terms of task space variables, thus establishing a direct mapping between state and control input. After training, an open-loop predictive controller is developed, which is implemented via trajectory optimization and iterative sequential quadratic programming. In [104], the approach is further extended to the implementation of a closed-loop controller.

In addition, reference [14] discusses the inverse kinematic modeling of a bionic trunk. In this study, the target is limited to a limited set of vertices during the learning phase, ensuring that the target vertex and the desired wrist joint position of the robot tip are within the specified volume range. The goal of the inverse model is to generate a posture that can move the effector to each vertex, and the training process continues to optimize until the distance between the target position and the actual position is minimized.

## 5. Discussion

The diverse modeling approaches for soft robotic arms reflect the multifaceted nature of their applications and underlying challenges. Numerical methods, despite their precision, often require substantial computational resources, making them more suitable for offline analysis and design optimization rather than real-time control. Analytical methods, with their simplicity and computational efficiency, provide a foundational understanding of soft arm behavior but struggle to account for highly nonlinear or dynamic interactions in complex environments. Meanwhile, data-driven approaches hold significant promise in bridging these gaps by learning system behaviors directly from data. However, their reliance on extensive datasets and potential overfitting to specific scenarios highlight the importance of robust data collection strategies and validation techniques.

The integration of these approaches offers an exciting direction for future research. For instance, hybrid models that combine the physical interpretability of analytical or numerical methods with the adaptability of data-driven approaches can potentially address the limitations of individual techniques. Furthermore, advancements in computational power and algorithms may reduce the trade-offs associated with high-fidelity numerical simulations, enabling their broader application in real-time scenarios. As soft robotics continues to expand into fields such as healthcare, underwater exploration, and industrial automation, the development of modeling strategies that balance accuracy, efficiency, and adaptability will be critical to unlocking their full potential.

## 6. Conclusions

This review highlights the advancements and challenges associated with modeling soft robotic arms through numerical, analytical, and data-driven approaches. Numerical methods provide high accuracy and are invaluable for detailed simulations, though their computational demands limit real-time applicability. Analytical methods offer efficient and interpretable models but often struggle with capturing the complex nonlinear dynamics of soft systems. Data-driven approaches, powered by artificial intelligence, present an emerging paradigm for adaptive and flexible modeling but face challenges in data quality, generalization, and physical interpretability.

Future research should focus on the development of hybrid approaches that combine the strengths of these methods. For instance, integrating data-driven algorithms with physically grounded numerical or analytical models can enhance both accuracy and efficiency. Moreover, advancements in computational technologies and machine learning techniques will further expand the possibilities for real-time control and complex system modeling. As the field of soft robotics continues to evolve, achieving a balance between precision, computational efficiency, and adaptability will be critical for advancing the design, control, and deployment of soft robotic arms in practical applications. For example, the use of fuzzy logic [105] and adaptive control methods can provide soft robots with a flexible and efficient solution in complex, uncertain and dynamic environments.

In recent research, we aim to design a modeling and control method that combines the advantages of Koopman operator theory and reinforcement learning. Koopman operator theory allows us to transform complex nonlinear systems into linear representations in a high-dimensional space, significantly enhancing the system’s modeling clarity and computational efficiency. Meanwhile, reinforcement learning excels at optimizing decision-making in dynamic environments, enabling the autonomous learning of optimal control strategies under uncertain or unknown conditions. By integrating these two approaches, the method not only enables precise modeling and efficient control of complex nonlinear systems but also provides remarkable resistance to external interference and adaptability to changing environments, offering a novel technical pathway and solution for the application of soft robots in complex scenarios.

## Figures and Tables

**Table 1:** Some simulators developed based on FEM.

Simulator	Title 2	Title 3
Abaqus	Fortran	Structural mechanics and material analysis
ANSYS	APDL	Multi-physics simulation and engineering analysis
COMSOL	C++	Multi-physics coupled simulation
ChainQueen	C++ and Python	Soft robotics and elastomer simulation
SOFA	C++ and Python	Medical Simulation and Soft Physics Simulation
TMTDyn	MATLAB	Multibody Dynamics Simulation
IPC	C++	Collision detection and physics simulation
AMBF	C++ and Python	Medical Robot Simulation and Training System
SOFiSTiK	C++	Structural Engineering and Bridge and Tunnel Simulation
